# Author Correction: An in situ activity assay for lysyl oxidases

**DOI:** 10.1038/s42003-021-02655-4

**Published:** 2021-09-16

**Authors:** Huilei Wang, Alan Poe, Lydia Pak, Kavitha Nandakumar, Sandeep Jandu, Jochen Steppan, Reik Löser, Lakshmi Santhanam

**Affiliations:** 1 Department of Biomedical Engineering, Johns Hopkins University, Baltimore, MD USA; 2 Department of Anesthesiology and Critical Care Medicine, Johns Hopkins University, Baltimore, MD USA; 3 Institute of Radiopharmaceutical Cancer Research, Helmholtz-Zentrum Dresden-Rossendorf, Dresden, Germany; 4 Department of Chemical and Biomolecular Engineering, Johns Hopkins University, Baltimore, MD USA

**Keywords:** Oxidoreductases, Biochemical assays, Mechanisms of disease, Oxidoreductases, Biochemical assays, Mechanisms of disease

Correction to: *Communications Biology* 10.1038/s42003-021-02354-0, published online 5 July 2021.

In the original version of the manuscript the authors had included an incorrect image in Fig. 3a (24 h, 50 μM) due to a copy and paste error. This has been corrected in the HTML and PDF versions of the article.

