# Supplementary material for: An in situ activity assay for lysyl oxidases
Source: Commun Biol. 2021 Jul 5;4:840. doi: 10.1038/s42003-021-02354-0 (PMC8257687; doi:10.1038/s42003-021-02354-0)
Supplement: Supplementary file 1 — Supplementary Information [file 42003_2021_2354_MOESM1_ESM.pdf]

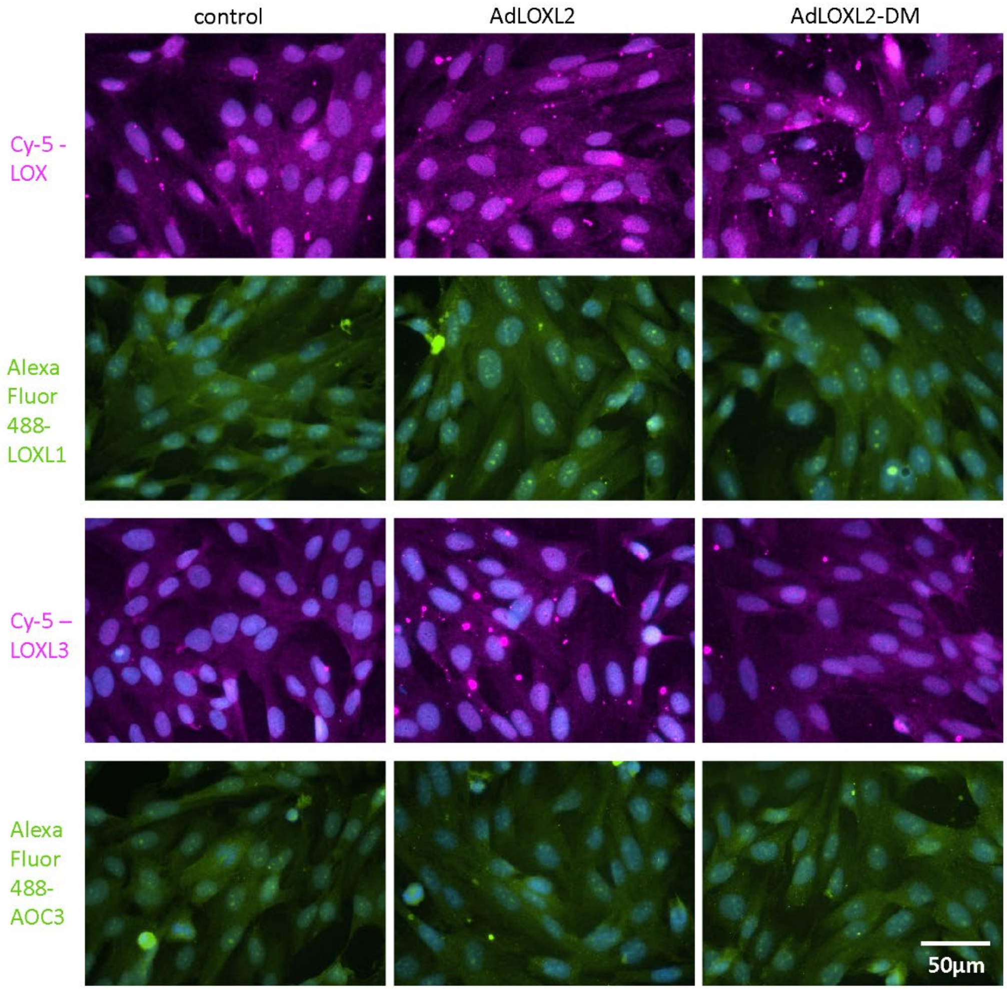

Figure S1: Representative immunofluorescence microscopy images of A7r5 cells transduced with AdLOXL2 or AdLOXL2-DM. *Cells overexpressing LOXL2 or LOXL2DM were stained for LOX, LOXL1, LOXL3 and AOC3 separately, and co-stained with DAPI (blue). No significant increase in expression was seen for these proteins in AdLOXL2 and AdLOXL2-DM cells. Images are representative of 6 independent experiments.*

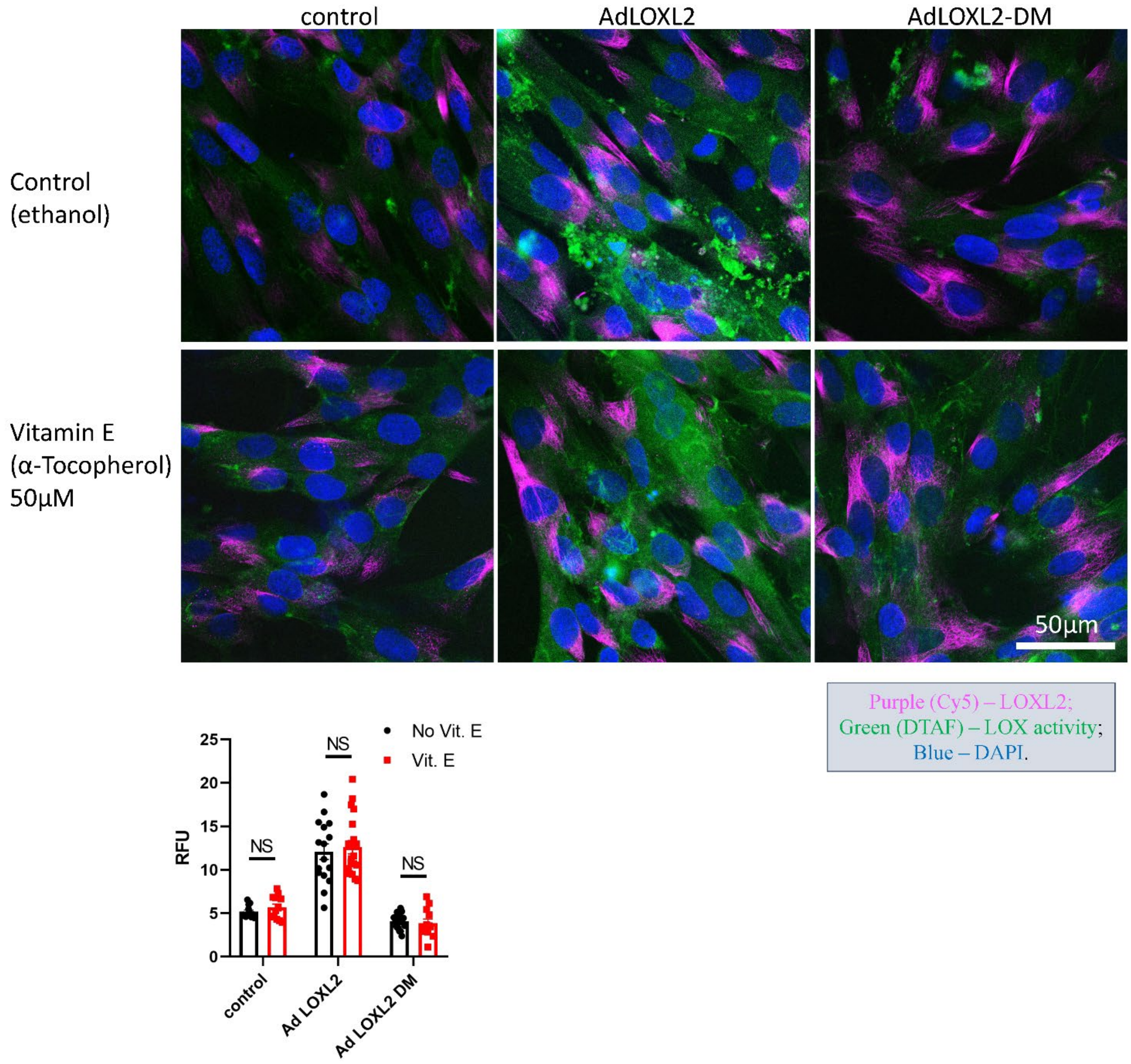

Figure S2: Representative confocal microscopy images of A7r5 cells transduced with AdLOXL2 or AdLOXL2-DM. *Cells were incubated with 100 μM biotin-hydrazide (BHZ), with or without vitamin E (50 μM α-Tocopherol) for 24 h and then fixed and co-stained for LOXL2 (purple; immunofluorescent staining), biotinylation corresponding to LOXs activity (green; DTAF-streptavidin), and nuclei (blue; DAPI). Activity signal in each IF image was converted to mean gray value shown in bar graph (n=12). BHZ*

*incorporation was unchanged in cells with the application of vitamin E. (n = 6; Scale bar = 50 μm. *P<0.05 by student's t-test.)*

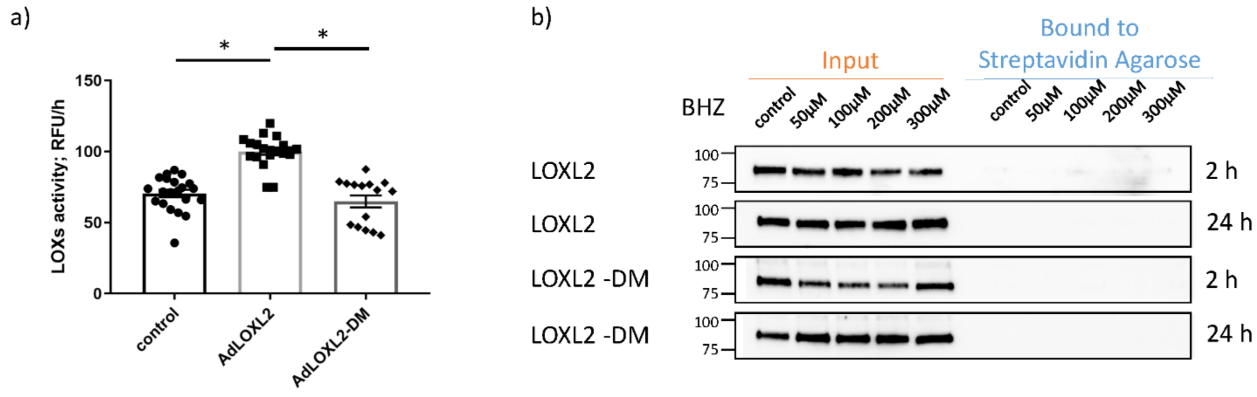

Figure S3. Biotin-hydrazide (BHZ) does not bind directly to LOXL2. a) *H_2O_2-resorufin coupled LOX activity assay in cell culture medium from A7r5 cells with and without adenoviral-induced overexpression of LOXL2 or LOXL2-DM Activity was calculated as the initial rate of reaction calculated as the slope of fluorescence (relative fluorescence unit (RFU)) per unit time (1 h) normalized to the average signal of AdLOXL2 samples. (n=15; *P<0.05 by student's t-test).* ***b)*** *Representative Western blot image (n=3) comparing total and biotinylated LOXL2 and LOXL2DM after incubation with increasing concentrations of BHZ for 2h or 24h followed by enrichment with Streptavidin agarose beads. Western blots showed no meaningful levels of biotinylated-LOXL2.*
