# Supplementary material for: An in situ activity assay for lysyl oxidases
Source: Commun Biol. 2021 Jul 5;4:840. doi: 10.1038/s42003-021-02354-0 (PMC8257687; doi:10.1038/s42003-021-02354-0)
Supplement: Supplementary file 2 — Description of Supplementary Files [file 42003_2021_2354_MOESM2_ESM.pdf]

**Description of Additional Supplementary Files**

**File name:** Supplementary Data 1
**Description:** All source data for graphs and charts.
