# Supplementary material for: An in situ activity assay for lysyl oxidases
Source: Commun Biol. 2021 Jul 5;4:840. doi: 10.1038/s42003-021-02354-0 (PMC8257687; doi:10.1038/s42003-021-02354-0)
Supplement: Supplementary file 4 — Reporting Summary [file 42003_2021_2354_MOESM4_ESM.pdf]

nature research

Corresponding author(s): Lakshmi Santhanam

Last updated by author(s): 06/04/2021

Reporting Summary

Nature Research wishes to improve the reproducibility of the work that we publish. This form provides structure for consistency and transparency in reporting. For further information on Nature Research policies, see Authors & Referees and the Editorial Policy Checklist.

Statistics

For all statistical analyses, confirm that the following items are present in the figure legend, table legend, main text, or Methods section.

n/a	Confirmed	
☐	☒	The exact sample size (*n*) for each experimental group/condition, given as a discrete number and unit of measurement
☐	☒	A statement on whether measurements were taken from distinct samples or whether the same sample was measured repeatedly
☐	☒	The statistical test(s) used AND whether they are one- or two-sided<br>*Only common tests should be described solely by name; describe more complex techniques in the Methods section.*
☐	☒	A description of all covariates tested
☐	☒	A description of any assumptions or corrections, such as tests of normality and adjustment for multiple comparisons
☐	☒	A full description of the statistical parameters including central tendency (e.g. means) or other basic estimates (e.g. regression coefficient) AND variation (e.g. standard deviation) or associated estimates of uncertainty (e.g. confidence intervals)
☐	☒	For null hypothesis testing, the test statistic (e.g. *F*, *t*, *r*) with confidence intervals, effect sizes, degrees of freedom and *P* value noted<br>*Give P values as exact values whenever suitable.*
☒	☐	For Bayesian analysis, information on the choice of priors and Markov chain Monte Carlo settings
☒	☐	For hierarchical and complex designs, identification of the appropriate level for tests and full reporting of outcomes
☒	☐	Estimates of effect sizes (e.g. Cohen's *d*, Pearson's *r*), indicating how they were calculated

Our web collection on statistics for biologists contains articles on many of the points above.

Software and code

Policy information about availability of computer code

Data collection	ZEN (2012 S4) was used to acquire confocal images on Zeiss LSM microscope. ChemiDoc MP was used to image western blots.
Data analysis	ImageJ was used to measure fluorescence intensity.<br>ImageLab (version 6.0.1) was used for western blot densitometry analysis. Plots and statistical analysis were generated with Graphpad Prism (version 8.0.1).

For manuscripts utilizing custom algorithms or software that are central to the research but not yet described in published literature, software must be made available to editors/reviewers. We strongly encourage code deposition in a community repository (e.g. GitHub). See the Nature Research guidelines for submitting code & software for further information.

Data

Policy information about availability of data

All manuscripts must include a data availability statement. This statement should provide the following information, where applicable:

- Accession codes, unique identifiers, or web links for publicly available datasets
- A list of figures that have associated raw data
- A description of any restrictions on data availability

The datasets generated during and/or analysed during the current study are available from the corresponding author on reasonable request.

Field-specific reporting

Please select the one below that is the best fit for your research. If you are not sure, read the appropriate sections before making your selection.

☒ Life sciences ☐ Behavioural & social sciences ☐ Ecological, evolutionary & environmental sciences

For a reference copy of the document with all sections, see nature.com/documents/nr-reporting-summary-flat.pdf

Life sciences study design

All studies must disclose on these points even when the disclosure is negative.

Sample size	All cell-based activity assays were performed on at least 3 coverslips and images were taken for at least 5 different random locations on each coverslip. We determined that this sample size was sufficient as staining variability was low. Replicate experiments were also conducted for additional data points.<br>For activity assays performed on aortic tissue isolated from mice, the sample size for each group was at least n = 6. Sample size was affected by the number of age-matched mice available at the time of the experiment.
Data exclusions	No data were excluded from this study.
Replication	Attempts at replication were successful. All data sets presented in the study combined results from independent attempts with appropriate normalization when possible.
Randomization	Aortic rings were selected randomly for exposure to biotin-hydrazide vs control.
Blinding	Blinding was not implemented in this study.

Reporting for specific materials, systems and methods

We require information from authors about some types of materials, experimental systems and methods used in many studies. Here, indicate whether each material, system or method listed is relevant to your study. If you are not sure if a list item applies to your research, read the appropriate section before selecting a response.

Materials & experimental systems

n/a	Involved in the study	
☐	☒	Antibodies
☐	☒	Eukaryotic cell lines
☒	☐	Palaeontology
☐	☒	Animals and other organisms
☒	☐	Human research participants
☒	☐	Clinical data

Methods

n/a	Involved in the study	
☒	☐	ChIP-seq
☒	☐	Flow cytometry
☒	☐	MRI-based neuroimaging

Antibodies

Antibodies used	LOXL2 rabbit monoclonal (Abcam, ab179810), LOX rabbit polyclonal (Invitrogen, PA1-46020), LOXL1 mouse monoclonal (Santa Cruz, sc-166632), LOXL3 rabbit polyclonal (Santa Cruz, sc-68939), VAP-1 (AOC3) mouse monoclonal antibody (Santa Cruz, sc-373924), Collagen I monoclonal antibody (Invitrogen, MA1-26771), Collagen IV polyclonal antibody (Assay Biotech, C0157), GAPDH mouse monoclonal (Novus Bio, catalog #NB300221), goat anti-mouse IgG (H+L)-HRP conjugate (Biorad 1706516), AffiniPure goat anti-rabbit IgG (H+L)-HRP conjugate (Jackson ImmunoResearch, 111035144), Cy5 AffiniPure Goat Anti-Rabbit IgG (H+L) (Jackson ImmunoResearch, 111175144), Alexa Fluor 568 Goat anti-Rabbit IgG (H+L) Secondary Antibody (Invitrogen, A-11011), Alexa Fluor 488 Donkey anti-Mouse IgG (H+L) Secondary Antibody (Invitrogen, A-21202) and fluorescein (DTAF) streptavidin (Jackson ImmunoResearch, 016010084)
Validation	Validated for WB and IHC by manufacturers.

Eukaryotic cell lines

Policy information about cell lines

Cell line source(s)	A7r5 (ATCC CRL1444)<br>HASMC (ThermoFisher C0075C)
Authentication	None of the cell lines used were authenticated.

Mycoplasma contamination	Cell lines were not tested for mycoplasma contamination.
Commonly misidentified lines (See ICLAC register)	N/A

Animals and other organisms

Policy information about studies involving animals; ARRIVE guidelines recommended for reporting animal research

Laboratory animals	Male LOXL2+/- and WT littermate mice were used in this study. Young group were less than 3 months and old group was greater than 18 months.
Wild animals	None
Field-collected samples	None
Ethics oversight	All animal protocols were approved by the Johns Hopkins Institutional Animal Care and Use Committee.

Note that full information on the approval of the study protocol must also be provided in the manuscript.
